# Gut microbiota and sepsis-associated acute kidney injury: a narrative review

**DOI:** 10.3389/fimmu.2026.1724266

**Published:** 2026-06-02

**Authors:** Hui Zhang, Bingling Fan, Ruze Ma, Ruowen Jiang, Zan Qin, Xueping Qu, Junwu Wang, Jiawei Xue, Caixia Wang, Xiaoqin Liu, Litao Guo

**Affiliations:** 1 Department of Critical Care Medicine, The First Affiliated Hospital of Xi’an Jiaotong University, Xi’an, Shaanxi, China; 2 Shaanxi Provincial Key Laboratory of Sepsis in Critical Care Medicine, Xi'an Jiaotong University, Xi’an, Shaanxi, China; 3 School of Clinical Medicine, Qinghai University, Xi'ning, Qinghai, China; 4 Department of Critical Care Medicine, Qinghai Provincial People's Hospital, Xi’ning, Qinghai, China

**Keywords:** acute kidney injury, fecal microbiota transplantation, gut microbiota, KPMP, probiotics, sepsis

## Abstract

**Background:**

Sepsis-associated acute kidney injury (SA-AKI) carries high morbidity and mortality, yet its pathogenesis remains incompletely understood. Emerging evidence underscores the gut-kidney axis as a critical pathway in SA-AKI development.

**Objective:**

This review aims to synthesize current knowledge on how sepsis-driven gut dysbiosis compromises intestinal barrier integrity and contributes to SA-AKI, and to explore potential therapeutic strategies targeting the gut microbiota.

**Methods:**

A comprehensive literature search was conducted in PubMed, Web of Science, and Scopus databases for publications between 2005 and 2026. Studies focusing on gut-kidney crosstalk mechanisms in sepsis/AKI were included. Key findings from human and animal studies were summarized.

**Results:**

Sepsis induces marked gut dysbiosis characterized by loss of microbial diversity and expansion of pathobionts. This dysbiosis compromises intestinal barrier integrity, facilitating translocation of bacterial products such as lipopolysaccharide (LPS). Upon entering circulation, these mediators activate systemic inflammation and renal signaling cascades, including the Toll-like receptor 4 (TLR4)/nuclear factor-kappa B (NF-κB) pathway, leading to tubular injury and impaired renal function. Recent human metagenomic studies have identified specific microbial signatures associated with AKI, such as increased *Clostridium asparagiforme* and decreased *Roseburia* spp., alongside elevated uremic toxin-producing bacteria like *Gordonibacter pamelaeae*. Additionally, gut-derived metabolites including indoxyl sulfate, p-cresol sulfate, and trimethylamine N-oxide (TMAO) have been implicated in promoting renal inflammation and fibrosis. Importantly, renal dysfunction further disrupts gut homeostasis, establishing a pathological gut–kidney feedback loop. Targeting the gut-kidney axis via fecal microbiota transplantation, probiotic supplementation, or short-chain fatty acid administration may offer novel therapeutic avenues.

**Conclusions:**

Sepsis induces gut microbiota dysregulation play an important role in the development of SA-AKI. The intestine-kidney crosstalk may provide a basis for the treatment of sepsis-induced organ injury and also provide new ideas for the treatment of SA-AKI.

## Introduction

Sepsis is a syndrome of organ dysfunction caused by an imbalance in the body’s inflammatory response caused by infection and is one of the leading causes of death in intensive care unit (ICU) patients (1, 2). Renal dysfunction is the most common organ injury in sepsis, namely sepsis-associated acute kidney injury (SA-AKI). The incidence of SA-AKI is approximately 40% to 50% and the mortality rate is as high as 70% in ICU septic patients (3, 4). Intestinal barrier dysfunction and disturbed gut microbiota are important contributors to the development and progression of sepsis. A large body of evidence suggests (5) that the gut microbiota is involved in the regulation of renal function in septic patients, but its pathophysiological mechanisms are not fully understood. The theory of the “gut–kidney axis” was first proposed in 2009 (6), and its core view is that during the progression of kidney injury, there is intestinal microecological disturbance, resulting in metabolic toxins produced by pathogenic bacteria entering the circulatory system, which triggers inflammatory responses and oxidative stress damage and further aggravates kidney injury. At the same time, these toxins cannot be removed by the damaged kidney in time, which exacerbates kidney injury and eventually forms a vicious cycle between the intestine and the kidney. The existence of the “gut–kidney axis” indicates bidirectional communication between the gut microbiota and the kidney. Therefore, this review discusses the changes of intestinal flora composition and the interaction between intestinal barrier disruption and SA-AKI in order to provide new ideas for clinical research and treatment.

## Methods

This narrative review systematically evaluates the role of gut microbiota in sepsis-associated acute kidney injury (SA-AKI) through the gut-kidney axis. A comprehensive literature search was conducted in PubMed, Web of Science, and Scopus databases for publications between 2005 and 2026, using the search strategy: (“sepsis” OR “septic shock”) AND (“acute kidney injury” OR “AKI”) AND (“gut microbiota” OR “dysbiosis”) AND (“gut-kidney axis” OR “short-chain fatty acids” OR “SCFAs” OR “fecal microbiota transplantation” OR “FMT” OR “uremic toxins” OR “indoxyl sulfate” OR “p-cresyl sulfate” OR “TMAO” OR “aryl hydrocarbon receptor” OR “AHR” OR “NF-κB” OR “chronic kidney disease” OR “CKD” OR “intestinal barrier” OR “leaky gut” OR “microbial metabolites”). After initial screening of titles and abstracts, studies meeting the following criteria were included: (1) original research (animal or human studies) or systematic reviews; (2) primary focus on gut-kidney crosstalk mechanisms in sepsis/AKI; (3) available full text in English. Case reports, conference abstracts, and non-English publications were excluded.

## Gut microbiota dysbiosis in kidney disease

The gut-kidney axis describes the bidirectional relationship between the intestinal microbiota and renal function (7). In chronic kidney disease (CKD), this axis becomes a key driver of pathology. Patients with CKD exhibit significant gut dysbiosis, characterized by a loss of beneficial commensals (e.g., SCFA-producing *Roseburia and Faecalibacterium*) and an overgrowth of pathobionts (e.g., *Escherichia coli*) *(*8, 9). This dysbiosis is driven by the uremic milieu itself; the influx of urea into the gut lumen favors the growth of bacteria expressing urease, which disrupts intestinal tight junctions and increases gut permeability—a condition known as “leaky gut” (7, 10, 11).

The consequences of this dysfunctional barrier are twofold. First, it facilitates the translocation of bacterial fragments like lipopolysaccharide (LPS) into the bloodstream, triggering systemic inflammation (11). Second, and more critically for kidney disease progression, it promotes the absorption of gut-derived uremic toxins, such as indoxyl sulfate (IS), p-cresyl sulfate (PCS), and trimethylamine N-oxide (TMAO) (12–14). These toxins, normally cleared by the kidneys, accumulate in the circulation when renal function is impaired. Once in the kidneys, they activate pro-inflammatory (e.g., NF-κB) and pro-fibrotic (e.g., aryl hydrocarbon receptor (AHR)) signaling pathways in renal tubular cells, directly contributing to tissue injury and fibrosis (12, 14–16). Simultaneously, the depletion of beneficial metabolites, particularly anti-inflammatory short-chain fatty acids (SCFAs), further compromises the intestinal barrier and amplifies systemic inflammation, creating a vicious cycle (16–18). Clinical studies confirm that elevated serum levels of IS and PCS are strongly associated with declining renal function and increased cardiovascular risk in CKD patients (19–21).

Thus, CKD is characterized by a self-perpetuating pathological loop: renal dysfunction fosters gut dysbiosis, and this dysbiosis, through the generation of uremic toxins and promotion of inflammation, in turn accelerates kidney damage. This pre-existing state of gut-kidney axis misalignment is crucial for understanding how an acute insult like sepsis can rapidly precipitate severe acute kidney injury (7, 22).

## Gut microbiota and sepsis

Most gut microbes belong to the following four major phyla: *Firmicutes*(also referred to as *Bacillota*), *Actinobacteria*, *Proteobacteria*, and *Bacteroidetes*(also referred to as *Bacteroidota*). *Bacteroidetes* and *Firmicutes* account for over 90% of all bacteria in the gut (23). Studies have shown that the ecological and functional microenvironment of the gut changes when sepsis develops (24–26). First, sepsis is associated with a significant reduction in both the diversity and richness of the gut microbiota. In animal models of septic shock induced by lipopolysaccharide, microbial α-diversity indices, such as the Shannon index, have been reported to decrease by approximately 30–50% (25). Corroborating these findings, clinical studies in patients with severe systemic inflammatory response syndrome (SIRS) demonstrate a marked decline in microbial richness. This is often characterized by a depletion of bacterial genera possessing anti-inflammatory properties, exemplified by *Faecalibacterium prausnitzii*, whose relative abundance typically plummets from approximately 5–10% in healthy individuals to less than 1% in severe cases (27). Second, sepsis precipitates a compositional shift in the gut microbiota. There is a substantial decrease in protective commensals, particularly obligate anaerobes and members of genera such as *Bifidobacterium* and *Lactobacillus*. For instance, fecal counts of Bifidobacterium may decline from approximately 10^9^ CFU/g in healthy subjects to below 10^5^ CFU/g in septic patients (5, 7). In addition, Hayakawa et al (28) delineated the acute temporal dynamics of gut flora post-insult, revealing that within six hours of a severe insult, counts of obligate anaerobes and Lactobacillus drop precipitously (e.g., Lactobacillus from ~10^6^ to <10³ CFU/g), while populations of bacteria including *Enterococcus* and *Pseudomonas* gradually increase over subsequent days. During this time, there were simultaneous decreases in three major short-chain fatty acids (SCFAs), including butyric acid, propionic acid, and acetic acid. Within 48 hours of the diagnosis of sepsis, intestinal microbiota abundance and bacterial diversity were reduced in septic patients. Importantly, another study found that the composition and functional components of the gut microbiota in septic patients have altered circadian rhythmicity (29). Current evidence suggests increased abundance of *Firmicutes* during the feeding phase and increased numbers of *Actinobacteria* and *Proteobacteria* during the fasting phase.

Notably, antibiotic therapy is the mainstay of treatment for sepsis and also leads to changes in the gut microbiota (30). Many broad-spectrum antibiotics inhibit or kill the dominant flora, resulting in increased colonization of opportunistic pathogens and fungi, ultimately leading to opportunistic or secondary infections and an exacerbated gut microbiome imbalance (31). An observational study using metagenomic sequencing, phylogenetic analysis, and microbial genome analysis confirmed that antibiotic use in the ICU significantly reduced diversity of the gut microbiota, and elimination of commensal strains promoted the spread of hospital-acquired *E. faecium* infection (32). The gut microbiome has been recognized as an important modulator of the host immune system, further complicating the use of antibiotics in the treatment of sepsis. For example, translocation of peptidoglycan from the gut can directly enhance neutrophil function and rapidly trigger systemic immunomodulatory responses to sepsis (33). Studies have also reported that intestinal symbionts can modulate systemic immunity (34). Specifically, antigens highly expressed on the outer membrane of gram-negative bacteria induce systemic serum immunoglobulin (Ig) G production, and this microbiome-specific IgG plays a protective role in systemic infection (34). Thus, the gut microbiota and its derived products have systemic effects on extraintestinal tissues and organs.

## Gut microbiota and SA-AKI

Changes in gut microbiota in sepsis are diverse and have some impact on the kidney. In 2009, Jang et al. (6) conducted experiments in germ-free mice demonstrating the connection between the gut and the kidney, and the basis of this theory is that renal injury leads to disturbance of the intestinal microbiota and impairs intestinal epithelial barrier function. In contrast, intestinal microecological imbalance can also produce metabolic toxins to aggravate renal injury. With the continuous improvements of genomic studies, the chain of “microbial toxin barrier inflammation” events in the intestine–kidney axis has been elucidated. First, sepsis itself impacts the abundance and composition of gut microbiota, thereby promoting the development and progression of acute kidney injury (AKI) (35). Interestingly, researchers have noted the renoprotective effect of gut-derived D-serine in AKI and elucidated the interaction between gut microbiota and kidney in health and AKI (36). Recent studies have also shown that SCFAs similarly play a protective role in AKI (37, 38). Paradoxically, Emal et al. (39) found that when broad-spectrum antibiotics were used to kill the intestinal microbiota in mice and renal ischemia-reperfusion (I/R) injury was performed, this apporach reduced renal injury, dysfunction, and remote organ injury and maintained tubular integrity after renal I/R injury. Mechanistically, depletion of gut microbiota prevented renal I/R injury through the maturation status of kidney-resident macrophages and monocytes (39). These two different mechanisms suggest that the gut microbiota has multiple effects on the kidney.

While animal models have provided foundational insights into gut–kidney crosstalk, translating these findings to human sepsis and SA-AKI requires careful consideration. Murine studies, such as those by Emal et al. (23), demonstrate that gut microbiota depletion can attenuate I/R-induced AKI, highlighting a context-dependent role of commensal microbes. However, human data from the Kidney Precision Medicine Project (KPMP) reveal a more complex picture. In a recent metagenomic whole-genome sequencing study of biopsy-proven AKI and CKD patients, distinct microbial signatures were identified compared to healthy controls, including increased *Clostridium asparagiforme* and decreased *Roseburia hominis* and *Roseburia intestinalis* in AKI (46). Notably, *Gordonibacter pamelaeae*—a known producer of p-cresyl sulfate—was elevated in both AKI and CKD, suggesting a potential role in uremic toxin accumulation (46). These findings contrast with some animal data, underscoring species-specific differences and the influence of clinical heterogeneity in human sepsis.

Alterations in the gut microbiota result in the release of inflammatory mediators into the blood circulation, which further impacts renal function. Druml et al. (40) proposed that the release of cytokines and inflammatory mediators increases oxidative stress, activation of various immune cells, neutrophil extravasation, and systemic renal injury. Studies using endotoxin-induced sepsis models have found that inflammatory mediators such as IL-1β, TNF-α, MCP-1, and NOS-2 levels are significantly increased in renal tissue (41). Li et al. (42) also showed that gut-derived endotoxicity is caused by increased intestinal permeability following severe renal I/R injury and subsequently amplifies the intrarenal inflammatory response by activating the renal of Toll-like receptor 4 (TLR4) signaling. In addition, alterations in gut microbiota lead to increased levels of oxidative stress in AKI and exacerbate ischemic injury. Second, sepsis disrupts the intestinal barrier and exacerbates the impact of the intestinal microbiota on the kidney. Sepsis leads to changes in integral and peripheral membrane proteins (i.e., myosin) that constitute tight junctions (TJs), resulting in a significant increase in intestinal permeability (43). Taken together, these studies suggest that damage to the intestinal mucosal barrier may stimulate pro-inflammatory events to occur, exacerbating the translocation of the gut microbial flora, which leads to kidney damage. AKI following sepsis also impacts the gut microbiota. A study using an AKI mouse model reported decreased abundance of *Bifidobacterium* and increased abundance of *Lactobacillus* and *Clostridium* following I/R injury (39). Permeability and integrity of the intestinal epithelial barrier is continuously compromised in AKI or following I/R injury, leading to spillage of bacteria, endotoxin, or macromolecules, exacerbating sepsis. In addition, Ranganathan et al (44) pointed out that metabolites produced by the gut microbiota lead to the accumulation of uremic toxins, such as indoxyl sulfate and p-cresol sulfate, leading to renal injury by promoting inflammatory infiltration and tubular atrophy. Gut-derived metabolites act as a critical bridge between the host and its microbiota, and among these, indoxyl sulfate (IS), p-cresyl sulfate (PCS), and trimethylamine N-oxide (TMAO) have emerged as key players in the pathophysiology of kidney diseases (13, 45). IS and PCS are generated exclusively by gut bacterial fermentation of dietary tyrosine and tryptophan, respectively. In the context of sepsis-induced gut dysbiosis and “leaky gut”, the production and translocation of these toxins are amplified (19). Once in the circulation, they bind extensively to albumin and are poorly cleared by the dysfunctional kidney. Mechanistically, IS and PCS are taken up into renal tubular cells via organic anion transporters (OATs), where they trigger oxidative stress, activate the NF-κB pathway, and stimulate the expression of pro-inflammatory and pro-fibrotic factors such as transforming growth factor-β1 (TGF-β1), thereby directly contributing to tubular injury and interstitial fibrosis (13). Elevated levels of these toxins in septic patients are strongly associated with the severity of AKI and adverse outcomes (46).

**Figure 1:**
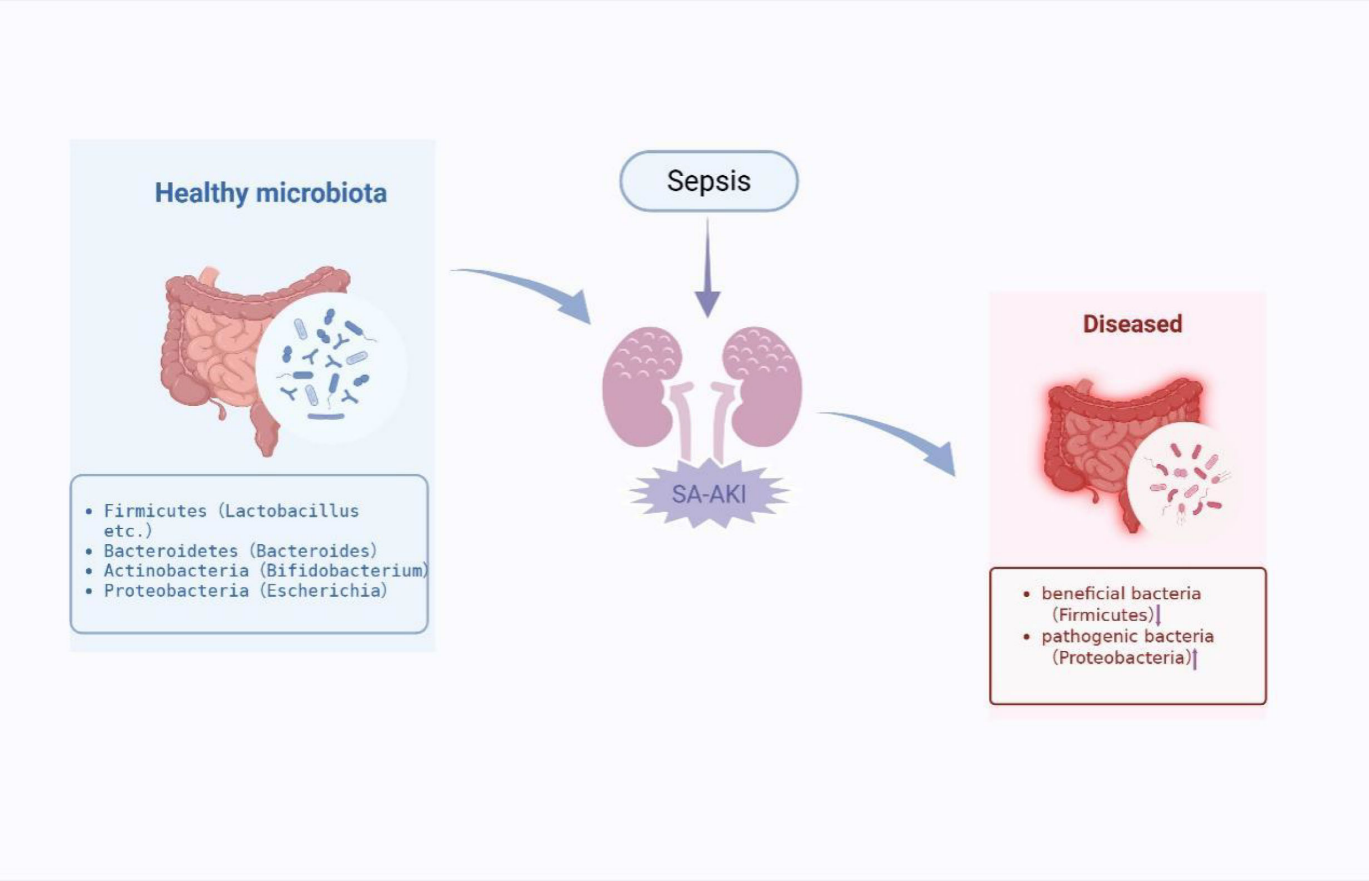
The microbiota composition of the major bacterial phyla;SA-AKI leads to a reduction in beneficial bacteria and a marked increase in pathogenic ones.

Similarly, TMAO, derived from the microbial metabolism of dietary choline and carnitine, has been implicated in renal damage. Elevated TMAO levels promote renal inflammation and fibrosis by activating the NLRP3 inflammasome and mitogen-activated protein kinase (MAPK) signaling pathways (45). In sepsis, the combination of increased microbial production and decreased renal clearance leads to a marked accumulation of TMAO, which correlates with worsened renal function and increased mortality (19). These metabolites exemplify how the gut microbiome can directly influence renal health, and their accumulation in SA-AKI represents a critical step in the self-perpetuating cycle of gut-kidney dysfunction. Taken together, the evidence indicates that sepsis-induced gut dysbiosis and barrier dysfunction are pivotal in the pathogenesis of SA-AKI. Building upon this association, the core mechanistic pathway translating gut disturbances to renal injury involves endotoxin translocation and the subsequent activation of renal immune-inflammatory signaling. (**Figure 1**).

**Figure 2:**
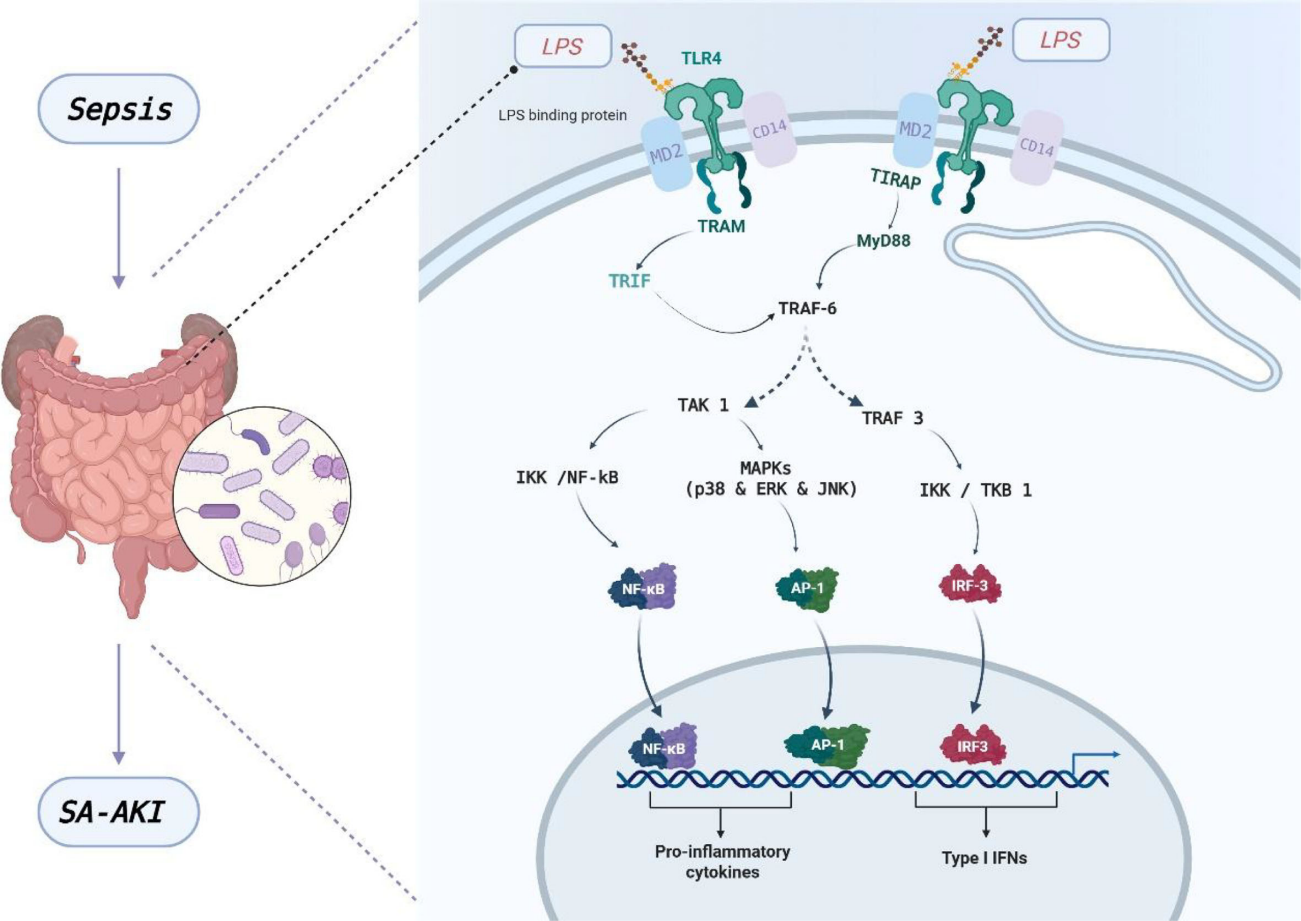
Intestinal-renal interaction in sepsis-induced acute kidney injury: toll-like receptor 4 (TLR4)-mediated tubular dysfunction. Lipopolysaccharide (LPS) binds to the extracellular domain of the TLR4/MD2 complex with the assistance of CD14, thereby activating both the MyD88-dependent and TRIF-dependent signaling pathways. Downstream signaling activates transcription factors such as NF-κB, AP-1, and IRF-3, which drive the expression of various pro-inflammatory genes, including cytokines, chemokines, adhesion molecules, and interferons. Concurrently, MAPK pathway kinases such as p38, ERK, and JNK are also activated. These disturbances in cellular signaling collectively lead to dysfunction in renal endothelial cells and tubules, alter local renal metabolism and hemodynamics, and ultimately contribute to the development of acute kidney injury.LPS (Lipopolysaccharide); TLR4 (Toll‐like receptor 4). TRIF (Toll/IL‐1 receptor domain containing adaptor inducing IFN‐β); IRF‐3 (interferon regulatory factor 3); NF-kB (nuclear factor kappa‐light‐chain enhancer of activated B cells);AP‐1 (activator protein 1); TRAF (TNF receptor associated factor);TAK1 (transforming growth factor beta‐activated kinase 1); TBK1 (tank binding kinase 1); IKK (I‐kappa B kinase complex); MAPKs (mitogen activated protein kinases).

Mechanistically, gut-derived pathogen-associated molecular patterns (PAMPs), particularly lipopolysaccharide (LPS), play a central role in this process. LPS translocates across the compromised intestinal barrier into the portal circulation and binds to Toll-like receptor 4 (TLR4) expressed on circulating immune cells (e.g., monocytes) and renal cells, such as renal tubular epithelial cells. This binding triggers conformational changes in the intracellular domain of TLR4, leading to the recruitment of the adaptor protein MyD88 and subsequent activation of the IκB kinase (IKK) complex. Activated IKK phosphorylates and degrades IκB, resulting in the release and nuclear translocation of nuclear factor-kappa B (NF-κB), which initiates the transcription of various pro-inflammatory cytokines and chemokines, including tumor necrosis factor-alpha (TNF-α), interleukin-1β (IL-1β), and monocyte chemoattractant protein-1 (MCP-1) (6, 47–49). Additionally, LPS-TLR4 signaling activates mitogen-activated protein kinase (MAPK) pathways (e.g., p38, JNK), further amplifying the inflammatory response. These factors not only directly damage renal tubular epithelial cells, inducing apoptosis and necroptosis, but also recruit and activate neutrophils and macrophages that infiltrate the renal interstitium. Through the release of reactive oxygen species (ROS) and proteases, they promote oxidative stress and exacerbate tissue injury, ultimately culminating in acute loss of renal function (**Figure 2**).

Current evidence is constrained by several limitations. Most mechanistic studies rely on rodent models, which may not fully replicate human sepsis pathophysiology or microbiome complexity. Human studies, including the KPMP, are often cross-sectional and feature small sample sizes—especially in AKI—limiting causal inference. Heterogeneity in sepsis etiology, antibiotic exposures, diet, and comorbidities further complicates interpretation. Moreover, while fecal microbiota transplantation (FMT) and probiotics show promise in animal and early human trials, their efficacy and safety in SA-AKI remain underexplored. The KPMP study also reported novel findings such as CrAssphage enrichment in AKI/CKD stools, yet the functional significance of this viral component is unknown (50). Beyond bacteria, the gut microbiome also contains archaea (e.g., Methanobrevibacter smithii) and viruses, both of which maintain host health. To date, research on their roles in CKD remains limited. In CKD, archaeal abundance negatively correlates with plasma TMAO (the “archaebiotic hypothesis”) (51), and the gut virome shows crAssphage enrichment and reduced NAD^+^ synthesis capacity (52). However, direct evidence in SA-AKI is lacking. Future longitudinal, multi-omics studies integrating microbial, metabolomic, and immunologic data are needed to clarify causality and identify targetable pathways in SA-AKI. These studies should expand to include non-bacterial commensals (archaea, viruses, fungi) using larger samples and archaea-specific detection methods to validate their causal roles in SA-AKI.

Notably, most primary studies included in this review did not stratify patients according to the Sepsis-3 criteria [i.e., based on Sequential Organ Failure Assessment (SOFA) score changes and vasopressor requirements]. Sepsis-3 defines septic shock as persistent hypotension requiring vasopressors to maintain mean arterial pressure ≥65 mmHg and serum lactate >2 mmol/L despite adequate volume resuscitation (1). Gut microbiota composition and its contribution to SA-AKI may differ substantially between septic shock and non-shock subgroups due to variations in hemodynamics, tissue perfusion, and antibiotic exposure. For instance, septic shock patients typically receive more aggressive fluid resuscitation and broader-spectrum antibiotics, both of which can profoundly alter gut microbial ecology. However, to date, no published study has specifically compared gut microbiota profiles between these two subgroups in SA-AKI patients. Therefore, future studies should explicitly adopt the Sepsis-3 framework and stratify patients by severity (septic shock vs. non-shock) to enable rigorous subgroup analyses of gut-kidney crosstalk in SA-AKI. (Key findings from human studies and another for rodent studies are summarized in **Tables 1**, **2**).

**Table 1:** Summary of human studies on gut microbiota in AKI, CKD and sepsis.

First author (year)	Title	Geographical area	Sample size	Population	Study aim	Key outcome/finding
Noel S et al. (2025) (50)	Metagenomic profiling of gut microbiota in Kidney Precision Medicine Project participants with CKD and AKI	USA (KPMP)	AKI/CKD patients	Biopsy-proven AKI and CKD patients	Metagenomic profiling of gut microbiota	Increased C. asparagiforme, decreased *Roseburia* spp. in AKI; elevated *G. pamelaeae* (p-cresyl sulfate producer) in both AKI and CKD; crAssphage enrichment
Corradi V et al. (2024) (19)	A possible role of p-cresyl sulfate and indoxyl sulfate as biomarkers in the prediction of renal function according to the GFR (G) categories	Italy	CKD patients	CKD patients at various stages	Evaluate IS and PCS as biomarkers of renal function	Both toxin levels significantly associated with GFR categories; potential as biomarkers for renal function prediction
Peng H et al. (2023) (21)	A metabolomics study of metabolites associated with the glomerular filtration rate	China	Multi-ethnic cohort	General population with GFR measurements	Metabolomics analysis of metabolites associated with GFR	Identified multiple gut-derived uremic toxins associated with declining GFR
Krukowski H et al. (2022) (7)	Gut microbiome studies in CKD: opportunities, pitfalls and therapeutic potential	Netherlands	CKD patients	CKD patients	Review of gut microbiome studies in CKD	Significant gut dysbiosis in CKD (loss of SCFA-producing *Roseburia* and *Faecalibacterium*, overgrowth of *E. coli*)
Xu Y et al. (2022) (23)	Contribution of gut microbiota toward renal function in sepsis	China	Sepsis patients	Septic patients	Role of gut microbiota in renal function during sepsis	Sepsis induces changes in gut microbiota diversity and composition, which in turn affects renal function
Ravi A et al. (2019) (32)	Loss of microbial diversity and pathogen domination of the gut microbiota in critically ill patients	UK	ICU critically ill patients	ICU patients receiving antibiotics	Metagenomic analysis of gut microbiota	Antibiotic use significantly reduced gut microbiota diversity; commensal elimination promoted hospital-acquired *E. faecium* spread
Glorieux G et al. (2021) (20)	Free p-cresyl sulfate shows the highest association with cardiovascular outcome in chronic kidney disease	Belgium	CKD patients	CKD patients at various stages	Free PCS and cardiovascular outcome	Free PCS shows the strongest association with cardiovascular outcomes in CKD
Hayakawa M et al. (2011) (28)	Dramatic changes of the gut flora immediately after severe and sudden insults	Japan	30 patients	Severe sudden insult (e.g., sepsis, trauma)	Acute temporal dynamics of gut flora	Within 6 hours: sharp decline in obligate anaerobes and *Lactobacillus*; within 48 hours: reduced microbiota abundance and diversity
Ranganathan N et al. (2010) (44)	Pilot study of probiotic dietary supplementation for promoting healthy kidney function in patients with chronic kidney disease	USA	CKD patients	CKD patients	Probiotic dietary supplementation	Oral probiotics reduced blood urea nitrogen levels
van Nood E et al. (2013) (70)	Duodenal infusion of donor feces for recurrent *Clostridium difficile*	Netherlands	RCT	Recurrent *C. difficile* infection patients	FMT vs. standard antibiotic therapy	FMT significantly more effective than standard antibiotics; increased fecal bacterial diversity in recipients
Shimizu K et al. (2006) (27)	Altered gut flora and environment in patients with severe SIRS	Japan	45 patients	Severe SIRS patients	Gut flora and environmental changes	Relative abundance of *F. prausnitzii* plummeted from 5-10% in healthy individuals to <1% in severe cases

**Table 2:** Summary of rodent studies on gut microbiota and AKI/SA-AKI.

First author (year)	Title	Animal model	Sample size	Intervention/exposure	Key outcome/finding
Miao H et al. (2024) (16)	Targeting *Lactobacillus johnsonii* to reverse chronic kidney disease	CKD mouse	Not specified	L*. Johnsonii* supplementation	Specific probiotic strain improves CKD progression by regulating gut microbiota
Li H-B et al. (2022) (17)	*Faecalibacterium prausnitzii* attenuates CKD via butyrate-renal GPR43 axis	CKD mouse	Not specified	F. *Prausnitzii* administration	Butyrate-producing bacteria exert renoprotective effects through renal GPR43 receptor
Luo L et al. (2022) (18)	Inulin-type fructans change the gut microbiota and prevent the development of diabetic nephropathy	Diabetic nephropathy rat	Not specified	Inulin-type fructans (dietary fiber)	Dietary fiber prevents kidney injury by modulating gut microbiota
Li J et al. (2019) (42)	Gut derived-endotoxin contributes to inflammation in severe ischemic acute kidney injury	Renal I/R injury mouse	8 per group	Renal I/R injury	I/R injury increases intestinal permeability; LPS amplifies intrarenal inflammation via TLR4 signaling
Zhu H et al. (2021) (61)	The probiotic *L. casei* Zhang slows the progression of acute and chronic kidney disease	Acute/chronic kidney disease mouse/rat	Not specified	*Lactobacillus* casei Zhang (probiotic)	Oral probiotic alleviates renal injury, improves gut dysbiosis and intestinal mucosal barrier damage
Nakade Y et al. (2018) (36)	Gut microbiota-derived D-serine protects against acute kidney injury	AKI mouse	12 per group	D-serine administration	Gut-derived D-serine exerts renoprotective effects; reveals gut-kidney interaction in health and AKI
Andrade-Oliveira V et al. (2015) (38)	Gut bacteria products prevent AKI induced by ischemia-reperfusion	Renal I/R injury mouse	Not specified	Short-chain fatty acids (SCFAs)	SCFAs prevent AKI through G-protein coupled receptors and HDAC inhibition
Emal D et al. (2017) (39)	Depletion of gut microbiota protects against renal ischemia-reperfusion injury	Renal I/R injury mouse	10 per group	Broad-spectrum antibiotics (gut microbiota depletion)	Microbiota depletion attenuated I/R-induced AKI via kidney-resident macrophage maturation
Jang HR et al. (2009) (6)	Early exposure to germs modifies kidney damage and inflammation after experimental ischemia-reperfusion injury	Germ-free mouse	Not specified	Early germ exposure	First demonstration of gut-kidney connection; germ-free mice showed altered renal injury severity
Huang H-C et al. (2021) (57)	Microbiota transplants from feces or gut content attenuated portal hypertension and portosystemic collaterals in cirrhotic rats	Cirrhotic rat	Not specified	Fecal microbiota transplantation (FMT)	FMT restored normal gut microbiota, downregulated phosphorylated eNOS, improved vasodilation
Li S et al. (2018) (69)	Intestinal microbiota impact sepsis associated encephalopathy via the vagus nerve	Septic rat	Not specified	Fecal microbiota transplantation (FMT)	FMT modulates gut microbial composition, increases commensals, reduces opportunistic pathogens, alleviates sepsis-associated encephalopathy
Dong F et al. (2022) (15)	Trimethylamine N-oxide promotes hyperoxaluria-induced calcium oxalate deposition and kidney injury by activating autophagy	Hyperoxaluria mouse	Not specified	TMAO administration	TMAO promotes renal crystal deposition and injury by activating autophagy
Liu Y et al. (2021) (37)	Fiber derived microbial metabolites prevent acute kidney injury through G-protein coupled receptors and HDAC inhibition	AKI mouse	Not specified	Short-chain fatty acids (SCFAs)	Fiber-derived microbial metabolites (SCFAs) prevent AKI through G-protein coupled receptors

## Gut–kidney interaction in SA-AKI

In the early phase of sepsis, the human body is in a state of hypermetabolization and accompanied by the release of a large number of pro-inflammatory mediators, which may present with decreased peripheral vascular resistance and increased cardiac output, resulting in increased renal blood flow and changes in glomerular filtration rate (23). Some scholars have found that gut microbiota is involved in this process and believe that there is a close relationship between the host and gut microbiota (53). The main physiological alterations during sepsis are the loss of integrity of the intestinal barrier and dysregulation of the intestinal microbiota.

**Figure 3:**
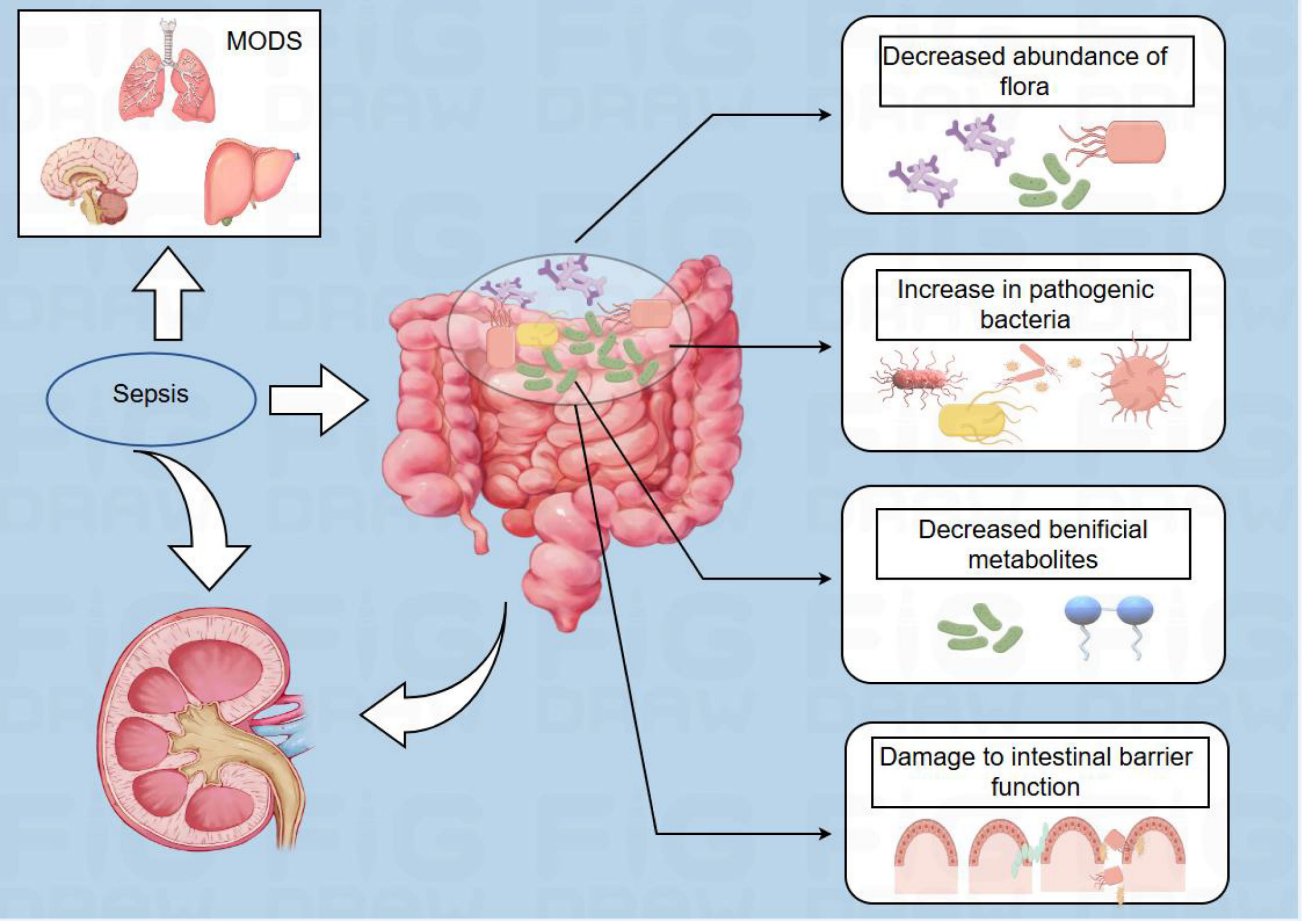
Gut–kidney interaction in SA-AKI. Sepsis can cause impairment of multiple organ function, with the development of sepsis, intestinal bacterial numbers and distribution, as well as microbial community structure, are altered, resulting loss of integrity of the intestinal barrier and dysregulation of the intestinal microbiota. It is also involved in the development of sepsis-associated acute kidney injury.

**Figure 4:**
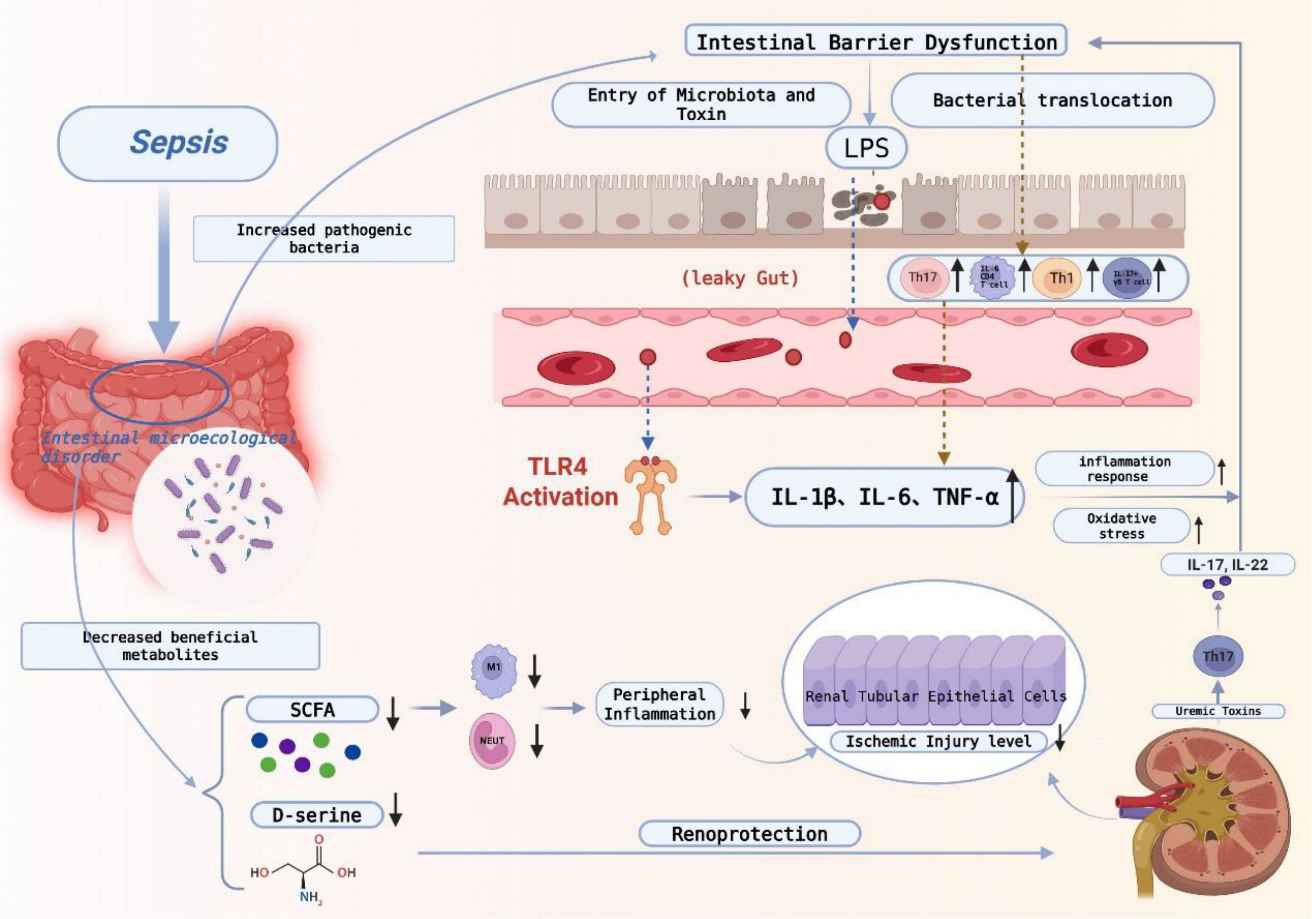
Schematic illustration of the vicious cycle in sepsis-associated acute kidney injury mediated by the gut-kidney axis. Short-chain fatty acids are transported via the systemic circulation to distant organs, where they attenuate peripheral inflammation by reducing the activation of macrophages, neutrophils, and other antigen-presenting cells. D-serine released by commensal bacteria crosses the intestinal barrier and exerts renoprotective effects in models of acute kidney injury. Disruption of the gut microbiota is characterized by the overgrowth of pathogenic bacteria at the expense of commensal species, leading to compromised intestinal barrier integrity and the translocation of bacteria and toxins into the systemic circulation (i.e., “leaky gut”). The dysbiotic gut microbiota releases excessive lipopolysaccharide, which enters the bloodstream through the impaired intestinal barrier. Lipopolysaccharide activates the pro-inflammatory functions of the immune system via the TLR4/nuclear factor-κB signaling pathway (e.g., IL-1β, IL-6, TNF-α), triggering systemic inflammation and enhancing oxidative stress. This further disrupts intestinal barrier function, exacerbating the leaky gut condition.

Intestinal epithelial cells not only promote food digestion and absorb nutrients (54), but also act as a physical barrier and can promote TJ formation between various cells. The TJ structure is mainly composed of transmembrane proteins [e.g., tight junction claudin, occludin, and junctional adhesion molecule-A (JAM-A)] and intracellular plaque proteins [e.g., zonula occludens (ZO) and cingulin]. Barrier function of the gut is able to prevent extraluminal escape of luminal contents and relies mainly on the presence of “TJ between luminal mask of intestinal epithelial cells and adjacent cells” and “JAM” (4). Damage of the intestinal barrier leads to increased permeability, and bacteria and toxins in the intestinal lumen enter the blood circulation through the intestinal wall. This damage subsequently causes the continuous production of inflammatory cytokines that can activate myosin light chain kinase and phosphorylate myosin light chain, resulting in the opening of TJs, further aggravating intestinal permeability and ultimately causing a “waterfall” cascade effect, which aggravates kidney injury (43, 55). Moreover, the number of bacterial species and total bacterial DNA are positively correlated with serum nitric oxide levels and negatively correlated with systemic vascular resistance when bacterial translocation occurs (56). Huang et al (57) found that fecal microbiota transplantation (FMT) restored normal gut microbiota composition and downregulated phosphorylated endothelial nitric oxide synthase, thereby improving vasodilation in rats. Therefore, the investigators hypothesize that gut microbiota in septic patients may promote increased renal blood flow by participating in systemic inflammatory response syndrome (SIRS), which may be a bidirectional synergistic effect between the gut and the kidney. Metabolites produced by the gut microbiota are similarly involved in the development of AKI. Nakad et al (36) showed that D-serine levels were significantly associated with decreased renal function in AKI patients, suggesting that gut-derived D-serine had a renoprotective effect. In summary, with the development of sepsis, intestinal bacterial numbers and distribution, as well as microbial community structure, are altered, resulting in an impaired intestinal barrier, strong inflammatory response, and reduced production of beneficial metabolites, which may play a key role in SA-AKI. (**Figures 3**, **4**).

It is important to recognize that the gut–kidney axis does not operate in isolation within the pathogenic network of SA-AKI. Intestinal dysbiosis and barrier dysfunction contribute to disease progression through endotoxin translocation and systemic inflammation, which in turn impair renal microcirculation and exacerbate oxidative stress. However, these processes coexist and interact with other hallmark mechanisms of sepsis-induced renal injury, including hyperdynamic/hypoperfusive hemodynamic disturbances, endothelial damage-mediated microcirculatory dysfunction, and activation of multiple programmed cell death pathways such as apoptosis, pyroptosis, and necroptosis (58–60). For instance, gut-derived inflammatory mediators can exacerbate vasodilation, reduce renal perfusion pressure, and directly trigger death-signaling cascades in tubular epithelial cells. Simultaneously, the accumulation of uremic toxins resulting from renal dysfunction further disrupts intestinal homeostasis, establishing a self-perpetuating vicious cycle. Thus, the gut–kidney axis represents a critical yet integrated component within the multifactorial pathogenesis of SA-AKI, interacting dynamically with hemodynamic, microcirculatory, and cellular death mechanisms to collectively drive disease progression.

### Preventative interventions

With the surge of research on the role of the gut microbiome in sepsis, its role in SA-AKI has also been gradually recognized. Based on the theory of the “gut–kidney” axis, treatments to correct intestinal microbiota disorders have become a research hotspot. Several potential preventive and therapeutic strategies to regulate the intestinal microbiota are available, which include supplementation of probiotics and SCFAs, selective digestive tract decontamination (SDD), and fecal bacteria transplantation.

### Supplementation with probiotics and short chain fatty acids

Probiotics may improve intestinal and immune homeostasis by restoring gut microbiota. A prospective study showed that oral probiotics reduced urea nitrogen levels (44). Zhu et al. (61) demonstrated that oral Lactobacillus casei relieves renal injury and can be used as a preventive intervention to improve intestinal flora disturbance, intestinal inflammation, and intestinal mucosal barrier damage caused by renal injury. In addition, many studies have shown that probiotics can improve immune function in septic patients by effectively reducing the inflammatory response (62, 63). SCFAs are products of dietary fiber that are metabolized by microorganisms, and the most studied SCFAs are butyrate, propionate, and acetate. SCFAs can regulate Treg cells, neutrophils, monocytes, and mast cells through G protein-coupled receptors, thereby regulating immune homeostasis in the body (64, 65). Treatment with SCFAs improved renal dysfunction in septic mice, mainly by regulating body immunity through expression involved in inflammatory processes (38).

### Antibiotic-based selective digestive tract decontamination

Since 90% of normal anaerobic flora is lost after sepsis develops, treating SA-AKI patients with SDD can/could reestablish the intestinal barrier, microbiome, and immune function. The aim of this intervention is to break the continuous cycle of damage and amplification of inflammation that occurs in the gut–kidney crosstalk pathway. In addition, a comprehensive systematic review and meta-analysis suggests that selective decontamination of the digestive tract with antibiotics prevents nosocomial infections and reduces overall mortality in critically ill patients respectively (66, 67). However, studies (53, 68) have found that the effects of different classes of antibiotics on the intestinal microbiota are largely unknown. It should be noted that according to the SIRS theory, the impact of early inflammatory response on the kidney is not all detrimental, and it is debatable whether aggressive use of broad-spectrum antibiotics to modify the gut microbiota increases the risk of kidney injury.

### Fecal microbiota transplantation

Microbial replacement therapy, particularly fecal microbiota transplantation, refers to the process of transplanting gut microbiota from healthy donors into patients, to reconstruct normal gut microecology. FMT exerts protective effects through anti-inflammatory and antioxidant mechanisms, as well as by supplementing the production of intestinal probiotic metabolites. An animal model study showed that FMT is able to modulate gut microbial composition, specifically acting to increase the abundance of commensals and reduce opportunistic enteric pathogens, relieving septic encephalopathy through anti-inflammatory effects (69). It has also been shown that FMT is significantly more effective than standard antibiotic therapy in the treatment of recurrent Clostridioides difficile infection by increasing the diversity of fecal bacteria in recipients (70). FMT has been successfully used to treat sepsis and improve clinical outcomes following alterations in microbiota composition and immune system (71, 72). FMT restores specific bacterial populations, promotes clearance of systemic pathogens and improves survival in sepsis. In addition, fecal bacteria transplantation was able to induce the expression of intestinal TJs and improve the survival rate of septic rats. Despite this evidence, so far, there have been few studies on the effects of FMT in SA-AKI. In addition, long-term safety trials on FMT are lacking, and some studies suggest that FMT is not always beneficial. Therefore, this research direction needs to be further explored in the future.

## Summary and prospects

As highlighted in this review, sepsis induces gut microbiota dysregulation play an important role in the development of SA-AKI. The intestine-kidney crosstalk may provide a basis for the treatment of sepsis-induced organ injury and also provide new ideas for the treatment of SA-AKI. Treatment strategies based on gut microbiota, such as probiotics, SDD, and FMT, have achieved some efficacy. Recent human metagenomic data from the Kidney Precision Medicine Project have further delineated specific bacterial and viral signatures in patients with biopsy-confirmed AKI and CKD, serving as a valuable high-resolution resource for biomarker discovery and mechanistic investigation (50).

However, current research on gut microbiota in SA-AKI remains limited, with most evidence derived from animal models and a scarcity of large-scale randomized clinical trials. Future studies should therefore prioritize early-phase (Phase I/II) clinical trials of fecal microbiota transplantation in SA-AKI, with emphasis on safety assessment—including risks of microbial mismatch and infection—and identification of efficacy biomarkers such as plasma short-chain fatty acid levels and inflammatory cytokine profiles. Such trials are essential to establish a robust theoretical foundation for elucidating the underlying mechanisms and advancing precision therapeutics targeting the gut microbiota in SA-AKI.
